# Direct laser writing of liquid crystal elastomers oriented by a horizontal electric field

**DOI:** 10.12688/openreseurope.14135.2

**Published:** 2021-11-19

**Authors:** Marco Carlotti, Omar Tricinci, Frank den Hoed, Stefano Palagi, Virgilio Mattoli

**Affiliations:** 1 Center for Materials Interfaces (CMI), Italian Institute of Technology, Viale Rinaldo Piaggio 34, Pontedera, 56025, Italy; 2 Engineering and Technology institute Groningen (ENTEG), University of Groningen, Nijenborgh 4, Groningen, 4747 AG, The Netherlands; 3 The Biorobotic Institute, Scuola Superiore Sant'Anna, Viale Rinaldo Piaggio 34, Pontedera, 56025, Italy

**Keywords:** Liquid Crystal Elastomers (LCEs), Microelectromechanical Systems (MEMS), Direct Laser Writing (DLW), Two-Photon Polymerization (2PP)

## Abstract

**Background:** The ability to fabricate components capable of performing actuation in a reliable and controlled manner is one of the main research topics in the field of microelectromechanical systems (MEMS). However, the development of these technologies can be limited in many cases by 2D lithographic techniques employed in the fabrication process. Direct Laser Writing (DLW), a 3D microprinting technique based on two-photon polymerization, can offer novel solutions to prepare, both rapidly and reliably, 3D nano- and microstructures of arbitrary complexity. In addition, the use of functional materials in the printing process can result in the fabrication of smart and responsive devices.

**Methods:** In this study, we present a novel methodology for the printing of 3D actuating microelements comprising Liquid Crystal Elastomers (LCEs) obtained by DLW. The alignment of the mesogens was performed using a static electric field (1.7 V/µm) generated by indium-tin oxide (ITO) electrodes patterned directly on the printing substrates.

**Results:** When exposed to a temperature higher than 50°C, the printed microstructures actuated rapidly and reversibly of about 8% in the direction perpendicular to the director.

**Conclusions: **A novel methodology was developed that allows the printing of directional actuators comprising LCEs via DLW. To impart the necessary alignment of the mesogens, a static electric field was applied before the printing process by making use of flat ITO electrodes present on the printing substrates. The resulting microelements showed a reversible change in shape when heated higher than 50 °C.

## Introduction

In recent decades, microelectromechanical systems (MEMS) have become a fundamental part of our technology and everyday life, playing a vital role in many diverse applications ranging from the automotive industry, to consumer electronics, the biomedical, and apparels sectors ^1^. The downsizing of control and processing units to small components – usually ranging from few millimeters down to hundreds of nanometers – allows the fabrication of complex and effective systems of sensors and actuators which guarantee reliable performances and fast responses.

In most cases, MEMS devices are fabricated employing integrated circuits (IC) technologies which rely on 2D or 2.5D methodologies such as lithography, chemical and physical deposition, dry and wet etching, and thermal treatment just to mention the most common ^2^. Whereas this has permitted this technology to achieve remarkable results, prototyping and optimization of IC technologies is expensive, both in terms of materials and equipment, and is generally slow.

Novel 3D microprinting techniques can help overcome these barriers, allowing fast prototyping and employing affordable materials ^3– 5^. In addition, they can enable the printing of fully 3D micro- and nanostructures of arbitrary complexity and shape ^6^, which is an interesting added value for MEMS fabrication. Among several approaches, Direct Laser Writing (DLW) based on two-photon polymerization technology, is a fully-3D microprinting technique which allows the fabrication of microstructures and architectures with resolutions down to 100 nm ^7^ and high speeds (in the order of tens of mm/s) ^8, 9^.

This technique usually makes use of a fs-pulsed near infrared laser (NIR; more commonly, 780 nm) which is focused by means of optical elements inside a photoresist which is transparent to the laser wavelength and polymerize at shorter wavelengths. In the focus spot, the intensity of the radiation is sufficiently high to trigger two-photon absorption, a non-linear optical process in which a molecule can absorb two photons simultaneously to get in an excited state ^10^. Such phenomenon can start the polymerization process which crosslink and solidify the material. By moving the laser focus in space, one can therefore obtain a 3D structure reliably and reproducibly. Nonetheless, despite these capabilities and the effort placed on research around DLW, to the best of our knowledge, only a few examples of actual MEMS fabricated (at least partly) using DLW can be found in the literature ^11– 13^.

Next to the 3D geometrical freedom during the printing process, DLW also offers the possibility of employing a large plethora of functional materials which confer smart and active properties to the final structures ^10^. For instance, responsive materials which can change their shape in response to a precise stimulus in a controlled and reproducible fashion, can act as actuators powered by light, heat, pH, solvent interaction, magnetic and electric fields ^10^. The combination of DLW and complex functionalization is often referred to as 4D printing. Several recent reviews well-describe the state-of-the-art approaches to prepare and characterize actuating elements ^14, 15^. Of particular interest are materials that provide directional actuation, which can confer anisotropic motion to the different parts and components ^16^.

One strategy to achieve such control consists in the use of a bilayer of materials with different Young moduli (which in DLW can be simply achieve by varying the degree of cross-linking) ^17^, limiting the shape change and thereby induce bending ^18^. The bilayer limits the geometrical freedom and for this reason materials that intrinsically provide directional actuation are of particular interest, as they can confer anisotropic motion to the different parts and components ^19^.

Liquid crystal elastomers (LCEs) are one of the most studied directional actuation materials because of their simple fabrication techniques, the possibility of using a vast array of molecules with different properties, and their power output ^20– 23^. LCEs are a class of polymeric materials obtained by the polymerization of rigid and rod-shaped liquid crystal (LC) molecules. If the monomer molecules are in a nematic (or smectic) phase while the polymerization takes place, the local order of the mesogens is partially retained in the final material. Then, upon heating, the loss of order creates a reversible shape change, shortening the elastomer along the director (and lengthening it along the other directions, as the shape change occurs at almost constant volume). The alignment of the LC moieties is crucial for the performances of the final device ^24, 25^. The former is usually achieved through surface rubbing ^26, 27^, surface patterning and functionalization ^28, 29^, mechanical stretching ^30^, polarized light ^31^, and electromagnetic fields ^32, 33^.

Examples of LCE microstructures printed trough DLW have been reported, where DLW can trigger the polymerization of LC monomers functionalized with acrylate groups ^25, 27, 34, 35^. Most of them employed rubbing or surface modification to induce the alignment of the LC monomers ^36– 40^. In this way, however, the fabrication of the substrates is time consuming and needs to be tailored to the specific structure that one wants to print, limiting to a certain extent the rapid prototyping capability offered by DLW and preventing the use of mass-manufactured substrates. For example, Li *et al.* explored the tuning of alignment by a weak magnetic field using LCE’s cross-linked by diacrylates and applied them as light-intensity modulators, but did not combine the orientation method with 3D printing ^19^. 

One may overcome these issues by making use of electric fields to generate differently aligned phases within the same substrate: by placing electrodes accordingly, one may fabricate, in a single step, a series of actuators characterized by different properties, direction of actuation, and working conditions (even along their z-axis). Tartan *et al.* investigated in several studies the behavior of DLW-printed LCE structures in presence of a vertical electric field ^41, 42^. Very recently, Münchinger *et al.* showed the possibilities offered by a multi vector quasi-static electric field ^43^. Compared to the former – and also in combination with it –, the latter approach offers more practical possibilities for the fabrication of MEMS and functional structures, permitting the independent fabrication of actuators capable of produce motion in different directions within a single printing step. Notably, as we show in this study, horizontal alignment via an electric field, could also be achieved by employing flat electrodes. These latter can be printed or obtained through standard lithographic approaches directly on the substrate offering several advantages. For example, one can optimize the design of the substrate with minimum effort to match the requirement of the DLW printed devices by preparing a new design; at the same time, this approach offers a practical solution for scalability, since flat substrates with conductive paths on top can be easily mass produced. In addition, the possibility of preparing the electrodes directly on the printing substrate, makes this approach useful to be employed in most DLW machines, commercial or home built, as it does not require any extra modules. Moreover, as ultimate goal, this approach will make possible to reorient in real time the LCE during the DLW fabrication, allowing the creation of anisotropically aligned LCE domains in the same microstructures, thus allowing complex actuation patterns.

In this study, we report the use of DLW to fabricate several LCE microstructures to be used as actuators. We performed the alignment of the mesogens employing an electric field parallel to the substrate (1.7 V/µm) generated by applying bias to flat ITO electrodes prepared on the same glass substrate used for the printing process. The resulting structures were characterized by remarkable resolution and showed actuation when exposed to temperatures higher than 50 °C in analogy to macroscopic films prepared in the same manner.

## Methods

### Materials

The 2-methyl-1,4-phenylene bis(4-(3-(acryloyloxy)propoxy)benzoate) (RM257, LC-DA) and 4-((5-(acryloyloxy)pentyl)oxy)phenyl 4-methoxybenzoate (LC-MA) were obtained from SYNTHON Gmbh (Germany) and used as received. Photoinitiator Irgacure 369, chloroform, isopropanol, and hydrochloric acid 37% were obtained from SigmaAldrich (United Kingdom). Niric acid 75% was oobtained from CARLO ERBA Reagents (Italy, www.carloerbareagents.com). Indium-tin oxide (ITO)-coated glass slides (CEC020T, dimensions: 30±0.3, 0.175±0.015, resistance 10-20 Ω) were purchased from Tecnovetro (Italy). Photoresist AZ 10XT, developer and remover were acquired from Microchemicals Gmbh (Germany). Conductive silver paste (RS 186-3600) was obtained from RS Components (Italy). High voltage supply is obtained by using a P12P module by EMCO (France).

### Fabrication of indium-tin oxide interdigitated electrodes

ITO-coated glass slides were spuncoated with AZ 10XT (3400 rpm, 60 s, final thickness 10 µm) on a BLE Laboratory Equipment Delta 10 BM spincoater (Germany, b-l-e-laboratory-equipment.germanytrade.it) and soft baked at 120 °C for 100 s on a BLE Laboratory Equipment Delta 150 BM hotplate. A pattern of interdigitated electrodes was transferred onto the samples exposing them to UV radiation through a negative mask by means of a mask aligner (MA/BA6 SUSS MicroTec) employing the power dose suggested by the supplier (1500 mJ/cm ^2^). The exposed samples were developed for 20 minutes in AZ developer and rinsed thoroughly with water. Removal of the ITO was performed by immersion in freshly prepared aqua regia (1:3 HNO _3_ 75%:HCl 37%) for 10 s. After rinsing with abundant water, the mask was removed by immersion in AZ Remover for five minutes and rinsing with isopropyl alcohol. Cu tape was added to facilitate the wiring and were contacted using silver paste. The ITO electrodes used in this study were 200 µm wide and comprised a gap of 150 µm.

### Direct laser writing of liquid crystal elastomer structures

The calculated amount of photoinitiator to achieve 7%mol. was dissolved in 0.5 mL of chloroform and added to a mixture of LC-DA and LC-MA in a vial to form a homogeneous solution. The formulations employed in this study comprised LC-MA:LC-DA mixtures in 8:2, 7:3, and 6:4 molar ratios. An example for the preparation an 8:2 resist mixture is the following: 2.2 mg of Irgacure 369 were dissolved in 1 mL of chloroform through stirring, after complete dissolution, 0.5 mL of the latter solution were employed to dissolve 14.6 mg of LC-MA and 5.4 mg of LC-DA. The solutions were kept in the dark.

For the preparation of the substrate, the mixture was then dropcasted on the ITO electrodes, covered to prevent light damage to the photoresist, and heated to 90 °C on a hotplate until melting was complete. A DC bias was applied (1.7 V/µm) and the hotplate cooled to 30°C at a rate of 5 °C/20 minutes. The samples were then rapidly transferred to a Photonic Professional system GT2 ( Nanoscribe) which mounted a 780 nm laser to perform the printing process. For the latter, .stl files of the 3D models, consisting of pyramids, cubes cantilevers and square nails, were prepared using the free software Blender (v2.93) and processed for the 3D printing process by performing the slicing using the machine proprietary software ( DeScribe). Hatching and slicing were set at 0.2 µm and 0.3 µm respectively. An oil immersion method as described in the manual of the supplier was used, mounting a 63x lens. A laser power of 20 mW (40% of nominal 50mW full power) and a laser speed of 10000 µm/s (equivalent to a “power dose” of 0.2 J/mm) were employed. After the printing, the structures were developed for 10 minutes in a 1:1 chloroform:isopropylalcohol solution and let dry in air.

### Evaluation of actuation properties

A substrate bearing the printed structure was placed over a Peltri element under an optical microscope (Hirox KH-8700 digital microscope) and repeatedly heated up to 70 °C and cooled down to room temperature (5 cycles). Measurements were taken using the image processing software of the instrument (KH-8700 v1.40a, HRMT v1.04).

### Simulations

All simulations were carried out in COMSOL Multiphysics 5.6. The purpose of the simulations was to illustrate that the electric field is sufficiently homogeneous under our conditions, and to show that the behavior observed in the material is consistent with what is expected from the theory and thus from simulation. The COMSOL simulations are therefore not critical for the reproducibility of the methodology presented above. A suitable open software alternative to reproduce the calculation could be OPENFoam, however, the authors did not test it.

Electric field simulation: we performed a 2D simulation using the AC/DC Module of COMSOL. We simulated a 400 µm wide section of the electrodes structure a 100 µm thick silica glass layer (relative permittivity of 2.09), on top of which is the 100 µm thick LC layer (relative permittivity of 3). On top and bottom are two layers of air (infinite domains). The electrodes are modelled as line boundaries, each 100 µm wide, at the glass-LC interface, placed at the two sides of the domain (leaving a 200 µm spacing between them. The condition of zero charge is applied to all external boundaries; to each electrode is applied an electric potential (+dV/2 to the left one and –dV/2 to the right one, where dV = 1250 V).

LCE structures simulations: we performed 3D simulations of the cube and nail structures using the Structural Mechanics Module of COMSOL. The geometries are imported from the corresponding .stl files. The LCE is modelled as a Linear Elastic Material with Young's Modulus E = 1 MPa, Poisson’s ratio ν ~ 0.5 (nearly incompressible), and density ρ = 1.2 g/cm ^3^. As in Palagi *et al.*, ^44^ the thermal response of the LCE is simulated by defining the dependence of the order parameter q on temperature according to the function $q=Q_{n}{\left(1+e^{\frac{T-T_{ni}}{\gamma }}\right)}^{-1},$ where Q _n_ is the order parameter of the nematic phase, which we set to 0.1, T _ni_ is the nematic-to-isotropic transition temperature, which we set to 60°C, and γ is a parameters that defines the width in temperature of the transition (here we set γ = 3K) ^45^. The active stretch of the LCE is then defined as $\lambda ={\left(\frac{1+2q}{1-q}\right)}^{\frac{1}{3}}{\left(\frac{1+2Q_{n}}{1-Q_{n}}\right)}^{-\frac{1}{3}}.$ The strain along the director is then defined as $\varepsilon_{Z}=\frac{1}{2}(\lambda^{2}-1),$ whereas the strain along the two perpendicular directions is $\varepsilon_{XY}=\frac{1-\lambda }{2\lambda }.$ These strains, which thus depend on temperature, are set as initial strain to the material. The structures have a fixed constraint on the bottom boundary. The LCE response is simulated by sweeping the temperature T between 40°C and 80°C with a step of 5°C.

The simulation results in .csv format are available in *Extended data* ^46^ for further independent analysis and post processing.

## Results and discussion

All optical microscopy and scanning electron microscope (SEM) images presented in this section are available in *Underlying data* ^46^.

The combination of the performances of LCE actuators with the unparalleled structural freedom offered by DLW at the micro/nano scale, can offer novel fascinating opportunities for the fabrication of functional MEMS.

**Figure 1.**
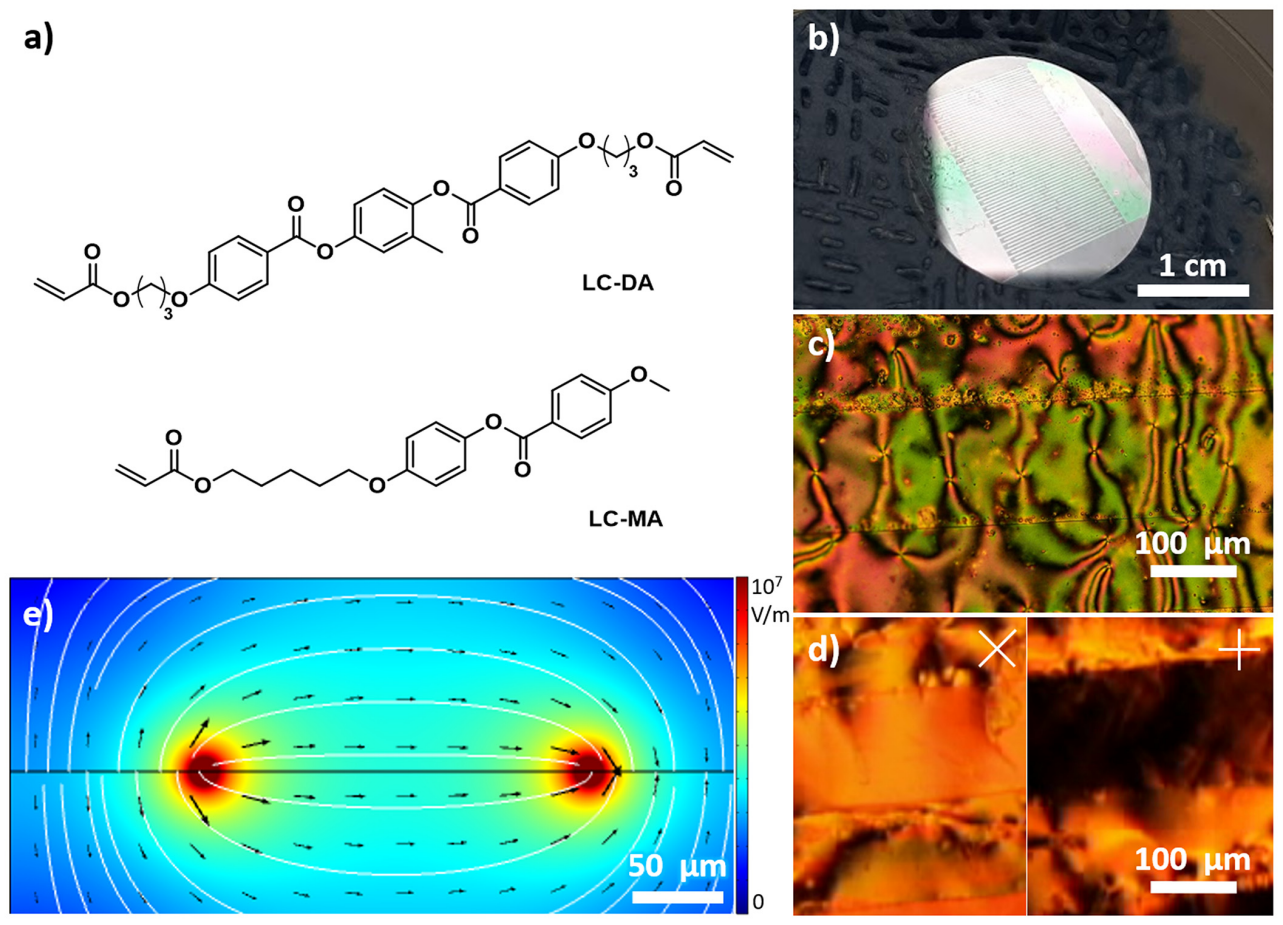
**a**) Molecular structures of the liquid crystal elastomers (LCE) monomers employed. **b**) Photograph of indium-tin oxide (ITO) interdigitated electrodes. **c**– **d**) Optical microscopy images of liquid crystals on substrate under cross polarizers no bias applied ( **c**) and with bias applied ( **d**; the white lines represent the orientation of the two polarizers). **e**) Simulation of the distribution of the electric field generated by flat electrode, view along the plane (cross section).

In order to fully exploit the potential of DLW, however, one must be able to orient LCEs to fit their needs, and that includes the possibility of orienting the mesogens in different directions across the substrate. To achieve this goal, flat electrodes (which can be easily prepared by lithographic approaches or evaporation) can be designed to carefully pattern the substrate and control the orientation of polar mesogens by application of an appropriate bias as they will tend to align to the applied electric field ^21^. While it is true that they cannot offer a homogeneous field perpendicular to the plane, its modulus only decreases of about 10% after 50 µm perpendicularly above the plane ( Figure 1), thus still allowing the fabrication of reasonably tall structures.

We designed our interdigitated electrodes to be 200 µm wide and characterized by a pitch of 150 µm. Such distance is much larger than what employed by Tartan *et al.* in their work in ref. 41 (5 µm), it however enables us to envision more flexible fabrication procedures for functional MEMS of dimensions of several tens of microns in the future. These electrodes (which served as substrate for the printing process directly) were fabricated from ITO-coated glass slides by application of a photoresist mask, etching of the exposed ITO surface, and removal of the mask. Electric contact was realized through copper tape and conductive silver paste.

The LCs we employed (LC-DA and LC-MA) are shown in Figure 1a and were chosen for their relatively large dipole moment and because they are commonly used by several research groups for DLW ^36, 39, 42^. When a melt comprising these compounds was cooled in the presence of an applied electric field of 1.7 V/µm, it tended to form large domains between the electrodes ( Figure 1c–d and Supporting VideoZ1 in *Extended data* ^46^). When we increased the bias beyond that threshold (e.g. 2.5 V/µm), we noticed the LC started to flow between the electrodes. Kuroboshi *et al.* recently described this phenomenon in a study about the behavior of electro-conjugate fluids between two electrodes at high bias ^47^. Notably, the application of a bias high enough to trigger this effect, was enough to provoke the melting of the mesogens from the solid phase. Deposition of a 100 nm dielectric layer (Parylene C) prevented LCs motion at high biases. Unfortunately, it also hindered dramatically the alignment of the mesogens which responded only to alternate fields and did not form large uniform domains.

**Figure 2.**
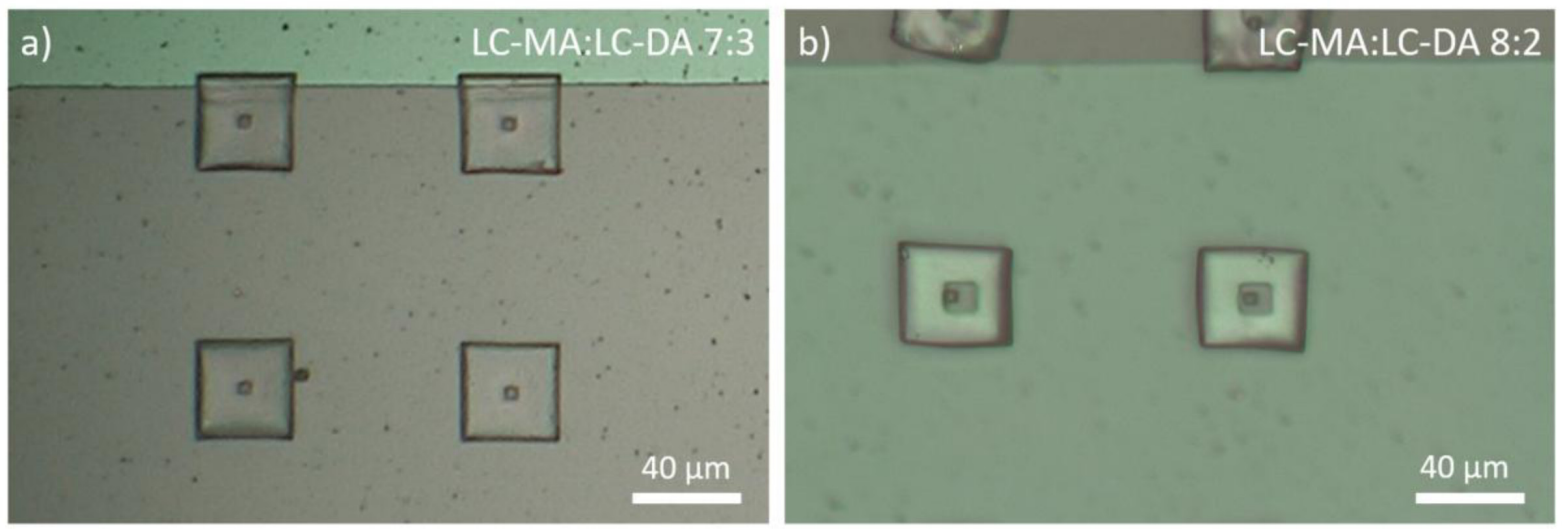
Optical microscopy images of micro nail structures realized using different formulations of 2-methyl-1,4-phenylene bis(4-(3-(acryloyloxy)propoxy)benzoate) (LC-DA) and 4-((5-(acryloyloxy)pentyl)oxy)phenyl 4-methoxybenzoate (LC-MA) : **a**) LC-MA:LC-DA molar ratio 7:3; **b**) LC-MA:LC-DA 8:2. An excess of monoacrylate component results in structures that look less defined and robust.

To align the mesogens and prepare the substrates for the microprinting process, a mixture comprising the LCs and a photoinitiator was placed on the electrodes as prepared and melted at 90 °C on a hotplate. We applied 250 V (1.7 V/µm) and allowed the sample to cool down slowly to room temperature (-15 °C/h). We tried several different compositions and found that the formulation comprising a molar ratio of 7:3 LC-MA:LC-DA gave the best results for our scope. In particular, when compared to different mixtures, the printed structures showed an appreciable resolution ( Figure 2) and resulted soft enough to observe the actuation (as discussed later and depicted in Figure 4). Increasing the amount of monoacrylate compound (8:2 LC-MA:LC-DA) resulted in structures that looked softer and less defined (see Figure 2), while increase of the quantity of difunctional LC (6:4 LC-MA:LC-DA) gave rise to more rigid structures that did not actuate appreciably.

**Figure 3.**
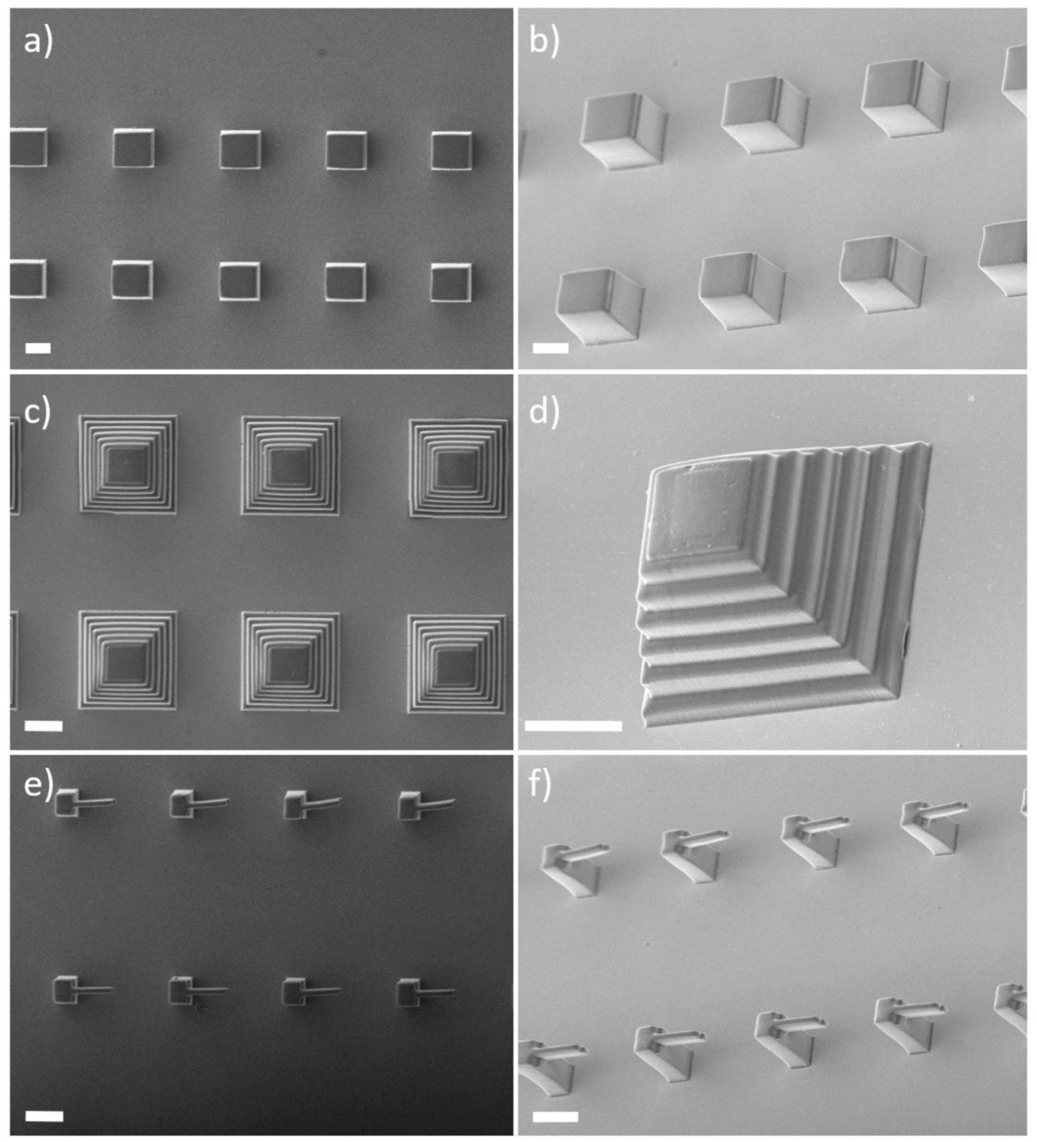
**a**, **b**) Scanning electron microscope (SEM) images (from above and sample tilted by 30°) of direct laser writing (DLW)-printed liquid crystal elastomers (LCE) microstructures comprising cubes, ( **c**, **d**), ‘Mayan’ pyramids, ( **e**, **f**), and cantilevers. Scale bars are 20 µm.

After several tests, the printing process was performed setting the laser power to 20 mW and the writing speed to 10 ^4^ µm/s (corresponding a power dose of 0.2 J/mm). Examples of microstructures (cubes, pyramids, and cantilevers) that it was possible to realize employing these conditions are shown in Figure 3. Remarkably, we observed minimal loss of focus and good resolution even for structures as tall as 50 µm despite the known birefringence of LC materials.

**Figure 4.**
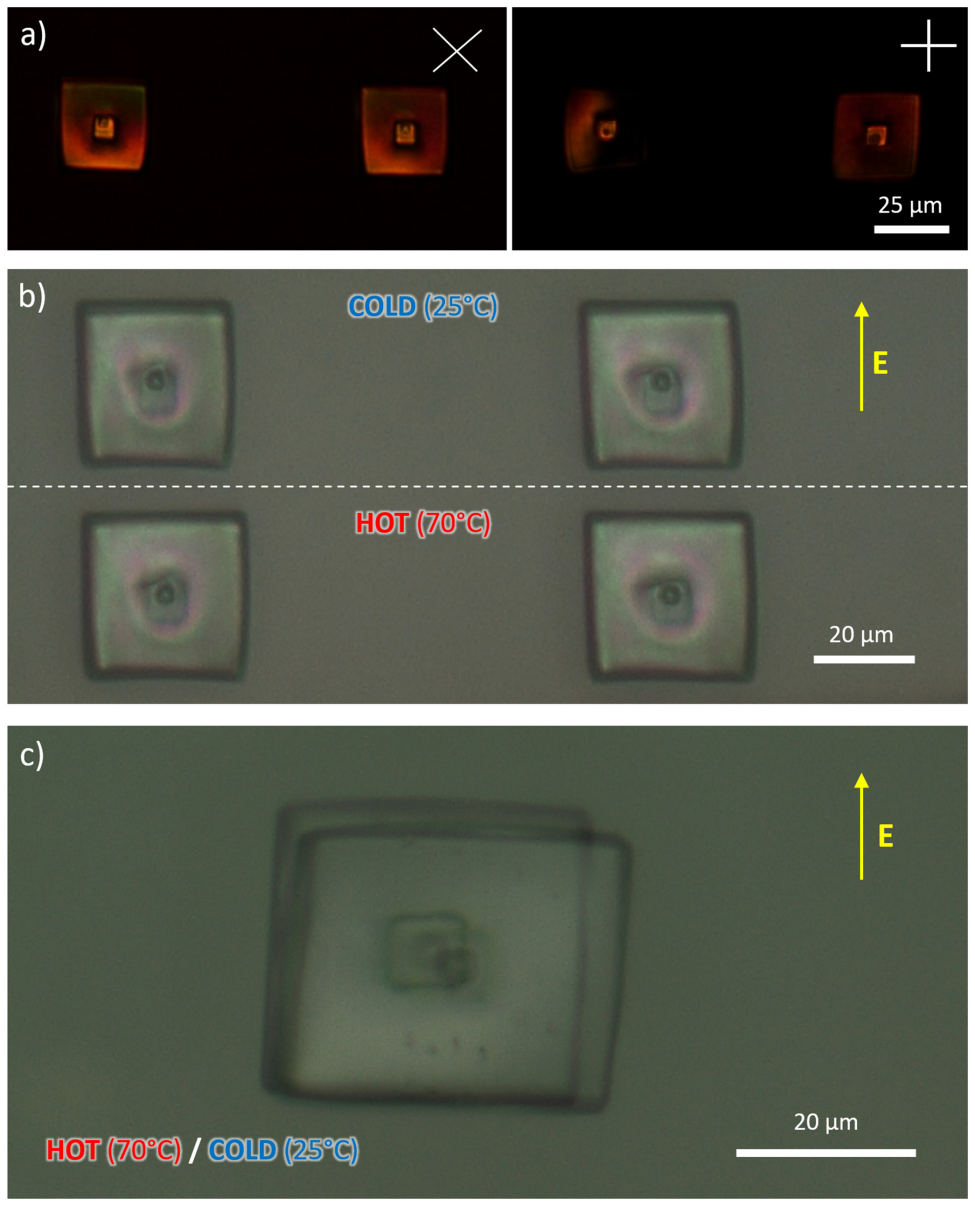
**a**) Optical microscopy images of micronails acquired through perpendicularly oriented polarizes placed at 45° and 0° with respect to the applied electric field. **b**) Example of thermally responsive actuation of nail structures: comparison of images recorded at room temperature and at 70 °C; **c**) and superimposed images of a different nail at the aforementioned temperatures.

**Figure 5.**
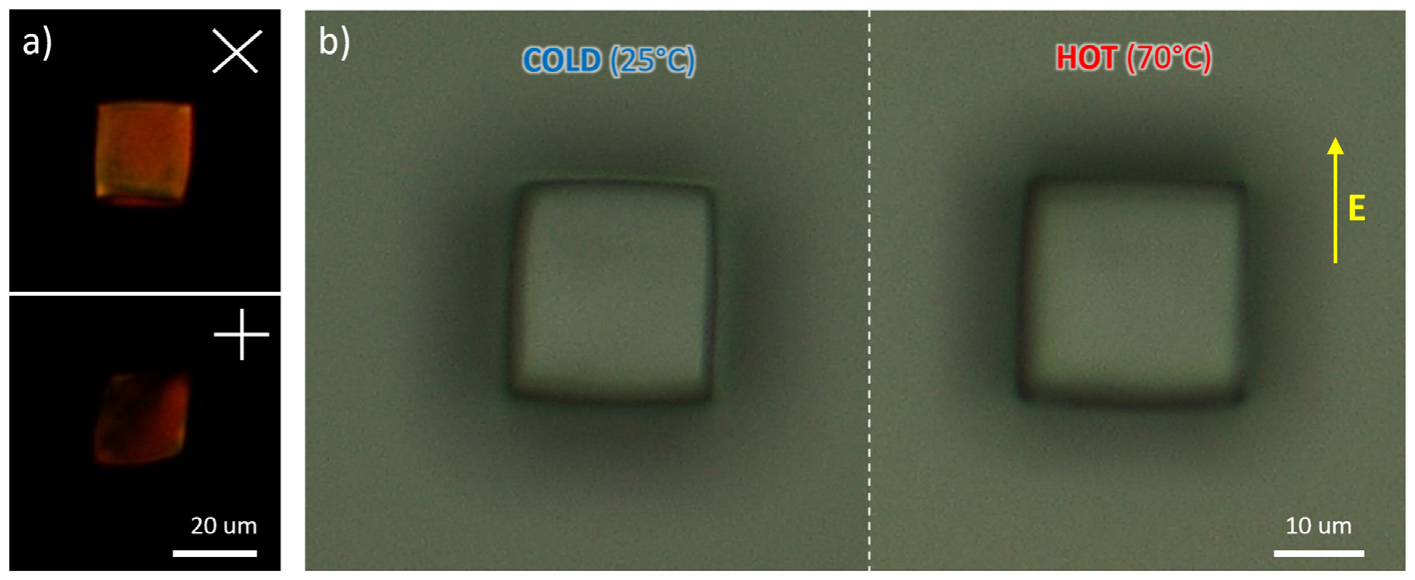
**a**) Optical microscopy images of microcubes acquired through perpendicularly oriented polarizes placed at 45° and 0° with respect to the applied electric field. **b**) Example of thermally responsive actuation via a comparison of images recorded at room temperature and at 70 °C.

To evaluate the actuation performances, we printed cubes and ‘square nails’ microstructures ( Figure 4 and Figure 5), which can deform in a predictable manner upon heating. Compared to the former, the nail is attached to the bottom of the substrate through a relatively small area, thus allowing a less constrained motion.

When perpendicular polarizers are used to observe these structures, they showed evidence of alignment along the direction of the electric field. Their arrangement was, however, not perfect, as one can see from the fact that the microstructures do not appear completely dark when one of the polarizers is parallel to the mesogens alignment direction. This observation could have been a consequence of the molecular structures and the composition of the formulation ^48^ (e.g. the presence of photoinitiator can affect the crystallinity) or a non-optimal alignment in the presence of the electric field generated by flat electrodes. It could also be a result of the high energy printing conditions typical of 2PP (which may disrupt the molecular packing locally).

It is worth mentioning that the printing process in the presence of a bias, produced structures that do not replicate exactly the input design but appeared more compressed in the direction perpendicular to the director. While we do not know the origin of such discrepancy, a similar phenomenon was observed by Münchinger *et al.*, ^43^ and could be related to the DLW printing process.

Upon heating to a temperature above 50 °C, both DLW-printed nails and cubes changed their shape, shortening along the director of about 2.8±0.5% and 1.9±0.5% respectively, and lengthening in the perpendicular direction of about 8.3±0.5% and 7.3±0.5% respectively ( Figure 4 and Figure 5, and Supporting VideoZ2; in *Extended data* ^46^ we also propose actuation of cantilevers, see Figure Z3). If we assume the lengthening along the z axis to be the same as the latter, we observe that the volume variation is not isovolumetric as it should be for LCEs ^44^. Currently, we do not have any hypothesis on why this happens. Studies are currently ongoing to determine whether it is an effect arising from the printing parameters or intrinsic to the printed microstructures.

 At about 70°C the actuation reached its maximum. Such deformations proceeded reliably and appeared to be fully reversible as it is common for LCE actuators. Indeed, as mentioned earlier, we found the degree of actuation to be larger for the nail structures compared to the solid cubes. The changes are, however, somewhat smaller than what can be expected from high-performance LCE systems ^20^. This could be due to the limited alignment obtained in the case of this study, which, according to our simulations, resulted in an ordering parameter of about 0.1 (see Supporting Video Z3 and Z4 in *Extended data* ^46^).

Unlike what reported by Münchinger *et al.*, ^43^ the actuation temperature was about 100 °C lower in our case (50 vs. 150 °C) despite the similar LC system employed. This difference could be related to higher amount of photoinitiator present in our formulation which can, in principle, affect the molecular characteristics of the crosslinked polymer by producing a larger number of shorter chains. This may affect several aspects of the final LCE, such as the overall crystallinity, the mechanical properties, and the transition temperature. Notably, the temperature of actuation was comparable to that of cm-long films prepared from an identical formulation and under the same electric field (see Figure Z2 in *Extended data* ^46^). This suggests that the properties of the material dominate the behavior of the devices even in the case of DLW-printed components.

## Conclusions

In this study, we showed a methodology for the preparation of directional LCE micro-actuators of arbitrary shape that can be printed via DLW and employed as active component in MEMS. We made use of a flat electrode pattern to align a photoresist formulation (comprising polar liquid crystal mesogens and a photoinitiator) using a static electric field of 1.7 V/µm. The resist was prepared by mixing together photoinitiator (7% mol.) and two mesogens characterized by a single and a double acrylate groups. The alignment of the molecules was obtained by melting the resist mixture on the patterned substrate and allowing it to slowly cool down to room temperature under a DC bias before undergoing the DLW process.

Despite the use of a flat pattern, simulations showed that the field generated decreased of less than 10% even at 50 µm from the substrate plane, and thus it allows the DLW printing process of structures with a reasonable height with little restrictions. The printed actuators could reliably and reproducibly actuate when exposed to temperatures higher than 50°C (full actuation at 70°C), with a measured maximum displacement of about 8%. These limited performances of DLW-printed LCEs compared to those prepared by more common methodologies could be possibly related to the non-ideal alignment of the LC molecules in the elastomeric matrix (we estimated an ordering parameter of 0.1).

We are currently working to better the design of the electrodes and the fabrication process in order to improve the yield and performances of the printed actuators with the final goal of integrating this technology in the fabrication of functional MEMS and microdevices. 

## Data availability

### Underlying data

Zenodo: Supporting information of paper "Direct Laser Writing of Liquid Crystal Elastomers Oriented by a Horizontal Electric Field". https://doi.org/10.5281/zenodo.5703137 ^46^.

This project contains the following underlying data within in the file ‘Underlying_data_Surce_Images.zip’:

- Fig1c-20X.tif (source picture of Figure 1, panel c: optical microscopy image of liquid crystals on substrate under cross polarizers no bias applied. Magnification 20X).
- Fig1d-10X.tif (source picture of Figure 1, panel d: optical microscopy image of liquid crystals on substrate under cross polarizers with bias applied. Magnification 10X).
- Fig2a.tif (source picture of Figure 2, panel a: optical microscopy images of different microstructures realized using a LC-MA and LC-DA formulation with 7:3 ratio).
- Fig2b.tif (source picture of Figure 2, panel b: optical microscopy images of different microstructures realized using a LC-MA and LC-DA formulation with 8:2 ratio).
- Fig3a.tif (source picture of Figure 3, panel a: SEM images of DLW-printed LCE cube microstructures, top view).
- Fig3b.tif (source picture of Figure 3, panel b: SEM images of DLW-printed LCE cube microstructures, 30° sample tilted view).
- Fig3c.tif (source picture of Figure 3, panel c: SEM images of DLW-printed LCE ‘Mayan’ pyramid microstructures, top view).
- Fig3d.tif (source picture of Figure 3, panel d: SEM images of DLW-printed LCE ‘Mayan’ pyramid microstructures, 30° sample tilted view).
- Fig3e.tif (source picture of Figure 3, panel e: SEM images of DLW-printed LCE cantilevers microstructures, top view).
- Fig3f.tif (source picture of Figure 3, panel f: SEM images of DLW-printed LCE cantilevers microstructures, 30° sample tilted view).
- Fig4a-Left20X.tif (source picture of Figure 4, panel a, left position: optical microscopy images of LCE micronails acquired through perpendicularly oriented polarizes placed at 45° with respect to the applied electric field. Magnification 20X).
- Fig4a-Right20X.tif (source picture of Figure 4, panel a, right position: optical microscopy images of LCE micronails acquired through perpendicularly oriented polarizes placed at 0° with respect to the applied electric field. Magnification 20X).
- Fig4b-Bottom.tif (source picture of Figure 4, panel b, bottom position: optical microscopy images of LCE micronails, example of thermally responsive actuation of nail structures: image at 70 °C).
- Fig4b-Top.tif (source picture of Figure 4, panel b, top position: optical microscopy images of LCE micronails, example of thermally responsive actuation of nail structures: image at room temperature).
- Fig4c-Hot.tif (source picture of Figure 4, panel c, hot frame of superimposed image: optical microscopy images of LCE micronails, example of thermally responsive actuation of nail structures: image at 70 °C. High magnification).
- Fig4c-Cold.tif (source picture of Figure 4, panel c, cold frame of superimposed image: optical microscopy images of LCE micronails, example of thermally responsive actuation of nail structures: image at room temperature. High magnification).
- Fig5a-Top20X.tif (source picture of Figure 5, panel a, top position: optical microscopy images of LCE cube acquired through perpendicularly oriented polarizes placed at 45° with respect to the applied electric field. Magnification 20X).
- Fig5a-Bottom20X.tif (source picture of Figure 5, panel a, bottom position: optical microscopy images of LCE cube acquired through perpendicularly oriented polarizes placed at 0° with respect to the applied electric field. Magnification 20X).
- Fig5b-Right.tif (source picture of Figure 5, panel b, right position: optical microscopy images of LCE cube, example of thermally responsive actuation of nail structures: image at 70 °C).
- Fig5b-Left.tif (source picture of Figure 5, panel b, left position: optical microscopy images of LCE cube, example of thermally responsive actuation of nail structures: image at room temperature).

### Extended data

Zenodo: Supporting information of paper "Direct Laser Writing of Liquid Crystal Elastomers Oriented by a Horizontal Electric Field". https://doi.org/10.5281/zenodo.5703137 ^46^.

This project contains the following extended data:

- Simulations.zip (Comsol Version 5.6 source simulation files for LCE microcube and LCE micronail upon heating, and for the electric field electric generated by flat electrode in experimental conditions. Complete Report file in PDF is also available, containing all the parameters and equations used for simulation. Finally, the simulation results are provided as text .csv files).
- SIVideo_LCAlignment.avi [Supporting VideoZ1] (video showing the response of the unpolymerized mesogens to an electric field of 1.7 V/µm).
- SIVideo_NailActuation.wmv [Supporting VideoZ2] (video showing the thermally activated actuation of a LCE nail microstructure upon several rt-75 °C cycles).
- SIVideo_SimluationCube.gif [Supporting VideoZ3] (video showing a simulation of the actuation of a LCE microcube upon heating).
- SIVideo_SimluationNail.gif [Supporting VideoZ4] (video showing a simulation of the actuation of a LCE micronail upon heating).
- ImageZ1.png [Supporting Figure Z1] (simulation of the distribution of the electric field generated by flat electrode, view along the plane, in cross section, with magnification of the central part of the working field).
- ImageZ2.png [Supporting Figure Z2] (photographs and thermal images of a LCE film obtained using a resin formulation identical to that employed in DLW on identical substrates that show the thermal response. Top: photograph of LCE films obtained by UV polymerization of a 7:3 LC-MA:LC-DA mixture over interdigitated flat ITO electrodes with an applied DC voltage. Upon heating with an infrared lamp (right column) the film bends and shortens. Middle: images acquired with a thermocamera highlighting the actuation temperature. Bottom: control LCE film polymerized as casted without electric field).
- “ImageZ3.png” [Supporting Figure Z3]. a) Optical microscopy images of double-cantilever structure acquired through perpendicularly oriented polarizes placed at 45° with respect to the applied electric field. b) Example of thermally responsive actuation via a comparison of images recorded at room temperature (upper) and at 70 °C (bottom).
- Files_Blender_LCE.zip (Blender Version 2.93 source file (.blend) for microstructure fabrication and generated surface .stl files, including cube, nail, cantilever and pyramid geometries).
- “Cube Describe.zip”. Example of DeScribe software output files containing all needed parameters used for printing cubes, generated starting from “cube.stl” file. Other geometries have been realised using the same writing parameters starting from appropriate “.stl” file.

Data are available under the terms of the Creative Commons Attribution 4.0 International license (CC-BY 4.0).
