# Supplementary material for: Direct laser writing of liquid crystal elastomers oriented by a horizontal electric field
Source: Open Res Eur. 2021 Nov 19;1:129. Originally published 2021 Oct 25. [Version 2] doi: 10.12688/openreseurope.14135.2 (PMC10445945; doi:10.12688/openreseurope.14135.2)
Supplement: Peer review history

# Peer review: Reviewer response for version 2

**DOI:** 10.21956/openreseurope.15449.r28065

**Published:** 2021-11-23

**Authors:** Daniele Martella

**Affiliations:** 1 Istituto Nazionale di Ricerca Metrologica INRiM, Turin, Italy

In the revised version, the authors improve both state-of-the-art, scope and discussion of the experimental results. I believe the article is now more clear and complete. Also non specialized readers can understand the findings of this research.

Is the rationale for developing the new method (or application) clearly explained?

Yes

Is the description of the method technically sound?

Yes

Are the conclusions about the method and its performance adequately supported by the findings presented in the article?

Yes

If any results are presented, are all the source data underlying the results available to ensure full reproducibility?

Yes

Are sufficient details provided to allow replication of the method development and its use by others?

Yes

Reviewer Expertise:

liquid crystal elastomer, direct laser writing, polymer chemistry, photoswitches

I confirm that I have read this submission and believe that I have an appropriate level of expertise to confirm that it is of an acceptable scientific standard.

# Peer review: Reviewer response for version 2

**DOI:** 10.21956/openreseurope.15449.r28064

**Published:** 2021-11-22

**Authors:** Ada-Ioana Ada-Ioana

**Affiliations:** 1 National Centre for Nano Fabrication and Characterization (DTU Nanolab), Technical University of Denmark, Lyngby, Denmark

The authors addressed the comments from myself and the other reviewer in a suitable manner. There are a few typos in the text that should be corrected, if possible.

Is the rationale for developing the new method (or application) clearly explained?

Partly

Is the description of the method technically sound?

Partly

Are the conclusions about the method and its performance adequately supported by the findings presented in the article?

Partly

If any results are presented, are all the source data underlying the results available to ensure full reproducibility?

No

Are sufficient details provided to allow replication of the method development and its use by others?

No

Reviewer Expertise:

Two-photon polymerization direct laser writing.

I confirm that I have read this submission and believe that I have an appropriate level of expertise to confirm that it is of an acceptable scientific standard.

# Peer review: Reviewer response for version 1

**DOI:** 10.21956/openreseurope.15240.r27908

**Published:** 2021-11-03

**Authors:** Daniele Martella

**Affiliations:** 1 Istituto Nazionale di Ricerca Metrologica INRiM, Turin, Italy

The authors describe the fabrication of DLW microstructures made by Liquid Crystalline Elastomers (LCEs). Thanks to the monomer alignment by electric field, the microstructures are able to deform under heating.

 The article is easy to understand and clear but before it passes peer review, I recommend a more detailed analysis of the state-of-the-art. Many applications have been already demonstrated for light driven or humidity responsive actuators (e.g. by Wiersma, Sitti and Schenning group, some relevant papers are already present in the article) and photonic devices (e.g. the following papers on which I am a co-author *ACS Photonics*, 2018, *5*(8), 3222-3230; *Advanced Optical Materials*, 2018, *6*(15), 1800167).

 Also the scope of the article has to be clarified. Since homogeneous planar alignment can be achieved with other simple techniques on large area (several cm), what are the advantages of the presented approach? What are the main differences with other setup that uses electric field for LC alignment?

 Other technical comment to improve the manuscript are listed below:

1. Introduction: “If the monomer molecules are in a smectic of nematic phase” has to be corrected in “If the monomer molecules are in nematic phase”. Both monomers form only nematic phase.
2. Figure 1: I suggest to explain better the POM figures in panel b and c since the alignment is a key point of this article. In particular, the author can show the homogeneous planar alignment by rotating the sample under the cross polarizer.
3. In the phrase: “We tried several different compositions and found that the formulation comprising a 7:3 LC-MA:LC-DA” what is the ratio 7:3? A mol/mol percentage? The author should cite the paper “ *Materials* **2016**, *9*(7), 525” talking about resolution with different crosslinker ratio.
4. Figure 3: the author should add the POM images of some 3D structures. Since they show poor alignment in the case of a cube, what is the resulting alignment in more complex structure (as the cantilever)?
5. Figure 4: even if the contraction is limited, the temperature decrease in the actuation with respect to previous articles is very interesting. By the way, the monomer composition is very similar to the ones previously reported and the alignment affects more the type and extent of deformation rather than the actuation temperature. The authors should clarify this point that, in my opinion, is related to the chemical structure of the polymer. The main difference with the literature is the very high amount of photoinitiator (7% mol/mol). I suggest the author to investigate how this parameter affect the deformation temperature (also in future studies).

Is the rationale for developing the new method (or application) clearly explained?

Yes

Is the description of the method technically sound?

Yes

Are the conclusions about the method and its performance adequately supported by the findings presented in the article?

Yes

If any results are presented, are all the source data underlying the results available to ensure full reproducibility?

Yes

Are sufficient details provided to allow replication of the method development and its use by others?

Yes

Reviewer Expertise:

liquid crystal elastomer, direct laser writing, polymer chemistry, photoswitches

I confirm that I have read this submission and believe that I have an appropriate level of expertise to confirm that it is of an acceptable scientific standard, however I have significant reservations, as outlined above.

## References

[1] : Three-Dimensional Photonic Circuits in Rigid and Soft Polymers Tunable by Light. *ACS Photonics* .2018;5(8) : 10.1021/acsphotonics.8b00461 3222-3230 10.1021/acsphotonics.8b00461

[2] : Structured Optical Materials Controlled by Light. *Advanced Optical Materials* .2018;6(15) : 10.1002/adom.201800167 10.1002/adom.201800167

[3] : Photoresist Design for Elastomeric Light Tunable Photonic Devices. *Materials (Basel)* .2016;9(7) : 10.3390/ma9070525 10.3390/ma9070525 28773646PMC5456896

# Response

**Published:** 2021-11-16

**Authors:** Virgilio Mattoli

**Affiliations:** Istituto Italiano di Tecnologia, Italy

**REPLY TO REVIEWER 2 COMMENTS** – (Reviewer’s comments in italics)

*The authors describe the fabrication of DLW microstructures made by Liquid Crystalline Elastomers (LCEs). Thanks to the monomer alignment by electric field, the microstructures are able to deform under heating.*

*The article is easy to understand and clear but before it passes peer review, I recommend a more detailed analysis of the state-of-the-art. Many applications have been already demonstrated for light driven or humidity responsive actuators (e.g. by Wiersma, Sitti and Schenning group, some relevant papers are already present in the article) and photonic devices (e.g. the following papers on which I am a co-author ACS Photonics, 2018, 5(8), 3222-3230; Advanced Optical Materials, 2018, 6(15), 1800167).*

**Author Reply:**  As already mentioned concerning the previous reviewer, we expanded the introduction adding the suggested papers and other DLW studies on LCEs and actuators.

*Also the scope of the article has to be clarified. Since homogeneous planar alignment can be achieved with other simple techniques on large area (several cm), what are the advantages of the presented approach? What are the main differences with other setup that uses electric field for LC alignment?*

**Author Reply:** The advantage concerning the use of flat electrodes over other – even simpler – methods consists in the possibility to provide selective (and not necessarily parallel) alignment in different region of the sample. By devising an appropriate electrode pattern, it is possible to print in a single printing step LCEs structures with different orientations. We are planning to use this approach in a follow up study about the fabrication of MEMS comprising LCEs actuators. Compared to other ways to apply electric fields, flat electrodes and lateral fields, can be prepared easily and with high throughput using standard lithographic techniques, they are easy to implement in the DLW machines, and do not pose any strong limit to height of the structures that can be build. Moreover, as ultimate goal, this approach will make possible to reorient in real time the LCE during the DLW fabrication, allowing the creation of anisotropically aligned LCE domains in the same microstructures, thus allowing complex actuation patterns. We made these points clearer in the introduction and in the text.

*Other technical comment to improve the manuscript are listed below: Introduction: “If the monomer molecules are in a smectic of nematic phase” has to be corrected in “If the monomer molecules are in nematic phase”. Both monomers form only nematic phase.*

**Author Reply: **The sentence was intended to refer to LC compounds in general, not limited to those used. ‘Smectic’ was therefore placed between brackets.

*Figure 1: I suggest to explain better the POM figures in panel b and c since the alignment is a key point of this article. In particular, the author can show the homogeneous planar alignment by rotating the sample under the cross polarizer.*

**Author Reply:** As the reviewer suggested, we added a picture of the same sample recorded with one polarizer aligned with the electric field.

*In the phrase: “We tried several different compositions and found that the formulation comprising a 7:3 LC-MA:LC-DA” what is the ratio 7:3? A mol/mol percentage? The author should cite the paper “Materials 2016, 9(7), 525” talking about resolution with different crosslinker ratio.*

**Author Reply:** The reviewer is indeed correct in pointing out that there was a missing detail. As they assumed, the ratio was indeed in moles. We added that information throughout the text.

*Figure 3: the author should add the POM images of some 3D structures. Since they show poor alignment in the case of a cube, what is the resulting alignment in more complex structure (as the cantilever)?*

**Author Reply: **Thin cantilever structures actuate less reliably, probably also because of the low young modulus, and were not included in the manuscript. We decided, however, to include an example in the Extended data as Figure Z3 (with reference in the main text). Unfortunately, the image we provided was recorded using only one configuration of the polarizers (45 deg) and we won’t be able to repeat the experiments anytime soon.

*Figure 4: even if the contraction is limited, the temperature decrease in the actuation with respect to previous articles is very interesting. By the way, the monomer composition is very similar to the ones previously reported and the alignment affects more the type and extent of deformation rather than the actuation temperature. The authors should clarify this point that, in my opinion, is related to the chemical structure of the polymer. The main difference with the literature is the very high amount of photoinitiator (7% mol/mol). I suggest the author to investigate how this parameter affect the deformation temperature (also in future studies).*

**Author Reply: **The reviewer suggested an interesting take that we did not consider while writing the manuscript. Indeed, the amount of photoinitiator present in the polymerized structures can affect several characteristics of the LCE such as the overall crystallinity, the mechanical properties, and the transition temperature. We included this thought in the main text, despite the fact that we currently cannot either prove or disprove the role of the photoinitiator in the lower actuation temperature compared to other studies. It still remains an interesting study for the future.

# Peer review: Reviewer response for version 1

**DOI:** 10.21956/openreseurope.15240.r27911

**Published:** 2021-10-29

**Authors:** Ada-Ioana Ada-Ioana

**Affiliations:** 1 National Centre for Nano Fabrication and Characterization (DTU Nanolab), Technical University of Denmark, Lyngby, Denmark

The article from Carlotti *et al. *describes the direct laser writing (DLW) of liquid crystal elastomer (LCE) microactuators which exhibit a reversible shape change of <10 % when actuated using heating (70C) / cooling (25C) cycles. The authors employed static electric fields to align the LCE mesogens before the DLW process and performed simulations to understand and verify their experimental results.

 DLW of LCE has been explored by various groups, so the novelty of the work is not very high, but it is reasonable for a Methods article. However, the experimental procedures are not described in sufficient details, especially for a Methods paper, and some of the data necessary to reproduce this work is missing. Furthermore, the introduction of the work is rather short, slightly misleading at times, and is missing some important articles from literature.

 I believe the article by Carlotti *et al.* is overall reasonably interesting and could be useful to the LCE DLW community. My detailed suggestions for improvement are listed below.

**Introduction**

 I believe that the introduction talks too much about MEMS and not enough about other relevant aspects, especially given the fact that the fabricated microactuators have potential for MEMS, but are not exactly MEMS by themselves. This is somewhat addressed in the Conclusions section. I suggest expanding the introduction to cover relevant aspect of DLW and reported applications of LCE patterned by DLW. Furthermore, I suggest mentioning that this paper is actually about 4D printing, since the fabricated structures are responsive to external stimuli.

 I suggest taking inspiration (and referencing) the following papers:

- https://doi.org/10.1002/aisy.202000256 and https://doi.org/10.1002/adom.201900156 - review papers covering microactuators prepared by LCE DLW.
- https://doi.org/10.1021/acsami.0c02781 ,  
https://doi.org/10.1002/aisy.202000035 , and https://doi.org/10.1021/acsapm.0c00626 - research articles on 4D printing by DLW
- https://doi.org/10.3390/micro1020013 - article about DLW on the Nanoscribe.

**Methods**

Materials - please add the product codes wherever possible, and include the purity of the chemicals. Please include all chemicals employed throughout the work (e.g. HNO _3_, HCl, isopropanol, chloroform, copper tape are all missing).

Fabrication of ITO IDE - Please add details on the equipment employed (spin coater, hotplate) and correct the repeated use of the word "washed".

DLW of LCE structures - “The calculated amount of photoinitiator to achieve 7%mol. was dissolved in 0.5 mL of chloroform” – please include the actual value in addition to this text. "(...) a mixture of LC-DA and LC-MA" - please list the ratios employed for preparing the various LC mixtures. There is a typo in "Calmar laser source". What is the model of Photonic Professional system? Is it a GT2? Finally and most importantly, I believe that the DeScribe files (data, job, recipe etc) need to be included in the Extended data, in addition to the .stl files which are already included.

Evaluation of actuation properties - “Peltri plate” – do the authors mean Peltier element? "repeatedly heated up to 70 °C and cooled down to room temperature" -  please include the number of heating / cooling cycles? "Measurements were taken using the image processing software of the instrument." - please provide the name and version of the software.

Simulations - in my opinion, the first paragraph of this section, with the exception of its first sentence, belongs in the Results and discussion section, rather than in the Methods section. How were the various parameters chosen? The authors mention is several places that xx is (...), "which we set to" yy (see Q _n_ , T _ni_ ). Similarly, "The LCE is modelled as a Linear Elastic Material with Young's Modulus E = 1 MPa, Poisson’s ratio ν ~ 0.5 (nearly incompressible), and density ρ = 1.2 g/cm ^3^." - how were the values chosen? Could the authors explain their choice or provide suitable references?

**Results and discussion**

 The caption for Figure 1b is extremely confusing. There is no clear separation between the description of b) and the combined description of c) and d). I suggest rewriting as " **a**) Molecular structures of the liquid crystal elastomers (LCE) monomers employed. **b**) Indium-tin oxide (ITO) interdigitated electrodes: view of substrate. ( **c,d**) Optical microscopy image of liquid crystals on substrate under cross polarizers, no bias applied ( **c**) and with bias applied ( **d**). **e**) Simulation of the distribution of the electric field generated by flat electrode, view along the plane (cross section)."

 When discussing the LC formulation, the authors selected "the formulation comprising a 7:3 LC-MA:LC-DA mixture" as optimal. First of all, could the reasoning behind this choice be described in more detail. Secondly, the authors then refer to the "6:4 LC-MA:LC-DA" mixture as having "an excess of the difunctional LC", although the LC-DA is only 40% of the mixture. Could the authors reconsider the way they refer to the mixtures that have different ratios that the one deemed optimal?

 In Figure 2 - please include **a)** and **b)** in the caption, since the images list this.

 In the caption of Figures 2-5 - please include **a)**, **b)**, **c)** etc before the corresponding text description, in a similar manner to the caption of Figure 1.

 In Figure 4a and Figure 5a, is it possible to provide images with higher magnification, or at least to crop the outer black area? It is rather difficult to observe anything in the current panels.

 As the authors mention, the alignment observed in the structures is sub-optimal. When addressing potential reasons, the authors do not mention the fact that an electric field induces a rather transient alignment, which might be lost during the DLW process. What is the fabrication time for the various structures? Have the authors investigated for how long after applying the electrical current do the mesogens stay aligned? The Methods section mentions that "The samples were then rapidly transferred to a Photonic Professional system" (after performing the alignment using the horizontal electric field). I believe that thoroughly characterizing the ability of the system to maintain its alignment over time, after the voltage source is removed, would be extremely beneficial, as the system could well lose its alignment over (relatively short) periods of time.

 Regarding the simulations and experiments: the heating / cooling cycles were 70C / 25C in the experiments, but 80C / 40C. Why is that? Would it not be more beneficial to employ the same temperature? In the Methods section, the authors mention that "T _ni_ is the nematic-to-isotropic transition temperature, which we set to 60°C". Why was this value chosen, and was it confirmed experimentally as well?

**Conclusions**

 This sections should be expanded. Since this is a Methods article, I believe the Conclusions section could briefly reiterate the process flow and emphasise crucial steps.

Is the rationale for developing the new method (or application) clearly explained?

Partly

Is the description of the method technically sound?

Partly

Are the conclusions about the method and its performance adequately supported by the findings presented in the article?

Partly

If any results are presented, are all the source data underlying the results available to ensure full reproducibility?

No

Are sufficient details provided to allow replication of the method development and its use by others?

No

Reviewer Expertise:

Two-photon polymerization direct laser writing.

I confirm that I have read this submission and believe that I have an appropriate level of expertise to confirm that it is of an acceptable scientific standard, however I have significant reservations, as outlined above.

## References

[1] : Light‐Powered Microrobots: Challenges and Opportunities for Hard and Soft Responsive Microswimmers. *Advanced Intelligent Systems* .2021;3(4) : 10.1002/aisy.202000256 10.1002/aisy.202000256

[2] : 3D Printed Photoresponsive Materials for Photonics. *Advanced Optical Materials* .2019;7(16) : 10.1002/adom.201900156 10.1002/adom.201900156

[3] : 4D Printing of a Light-Driven Soft Actuator with Programmed Printing Density. *ACS Appl Mater Interfaces* .2020;12(10) : 10.1021/acsami.0c02781 12176-12185 10.1021/acsami.0c02781 32073253PMC7135850

[4] : Color Modulation inMorpho Butterfly Wings Using Liquid Crystalline Elastomers. *Advanced Intelligent Systems* .2020;2(9) : 10.1002/aisy.202000035 10.1002/aisy.202000035

[5] : Direct Laser Writing to Generate Molds for Polymer Nanopillar Replication. *ACS Applied Polymer Materials* .2020;2(8) : 10.1021/acsapm.0c00626 3632-3641 10.1021/acsapm.0c00626

[6] : Micro 3D Printing by Two-Photon Polymerization: Configurations and Parameters for the Nanoscribe System. *Micro* .2021;1(2) : 10.3390/micro1020013 164-180 10.3390/micro1020013

# Response

**Published:** 2021-11-16

**Authors:** Virgilio Mattoli

**Affiliations:** Istituto Italiano di Tecnologia, Italy

**REPLY TO REVIEWER 1 COMMENTS** (Reviewer comments in italics)

*The article from Carlotti et al. describes the direct laser writing (DLW) of liquid crystal elastomer (LCE) microactuators which exhibit a reversible shape change of <10 % when actuated using heating (70C) / cooling (25C) cycles. The authors employed static electric fields to align the LCE mesogens before the DLW process and performed simulations to understand and verify their experimental results. DLW of LCE has been explored by various groups, so the novelty of the work is not very high, but it is reasonable for a Methods article. However, the experimental procedures are not described in sufficient details, especially for a Methods paper, and some of the data necessary to reproduce this work is missing. Furthermore, the introduction of the work is rather short, slightly misleading at times, and is missing some important articles from literature. I believe the article by Carlotti et al. is overall reasonably interesting and could be useful to the LCE DLW community. My detailed suggestions for improvement are listed below.*

**Author Reply:** We thank the reviewers for the time dedicated to our work. We incorporated their suggestions when it was possible and we hope that in this way the quality of the manuscript has improved. Below we address all the points raised.

*Introduction I believe that the introduction talks too much about MEMS and not enough about other relevant aspects, especially given the fact that the fabricated microactuators have potential for MEMS, but are not exactly MEMS by themselves. This is somewhat addressed in the Conclusions section. I suggest expanding the introduction to cover relevant aspect of DLW and reported applications of LCE patterned by DLW. Furthermore, I suggest mentioning that this paper is actually about 4D printing, since the fabricated structures are responsive to external stimuli. I suggest taking inspiration (and referencing) the following papers: https://doi.org/10.1002/aisy.202000256 and https://doi.org/10.1002/adom.201900156 - review papers covering microactuators prepared by LCE DLW. https://doi.org/10.1021/acsami.0c02781 ,  https://doi.org/10.1002/aisy.202000035 , and https://doi.org/10.1021/acsapm.0c00626 - research articles on 4D printing by DLW https://doi.org/10.3390/micro1020013 - article about DLW on the Nanoscribe.*

**Author Reply:** We thank the reviewer for the suggestions which was also raised by the second reviewer. We added several recent references to recent work and relevant reviews in version 2.

*Methods Materials - please add the product codes wherever possible, and include the purity of the chemicals. Please include all chemicals employed throughout the work (e.g. HNO3, HCl, isopropanol, chloroform, copper tape are all missing).*

**Author Reply:** Where possible we added such information.

*Fabrication of ITO IDE - Please add details on the equipment employed (spin coater, hotplate) and correct the repeated use of the word "washed".*

**Author Reply:** We added the missing information and fixed the repetition of the word ‘washed’.

*DLW of LCE structures - “The calculated amount of photoinitiator to achieve 7%mol. was dissolved in 0.5 mL of chloroform” – please include the actual value in addition to this text."(...) a mixture of LC-DA and LC-MA" - please list the ratios employed for preparing the various LC mixtures.*

**Author Reply: **Molar values were used as we did not always prepare the same amount of photoresist. However, we included an example recipe for those who are not familiar with the use of molar quantities.

*There is a typo in "Calmar laser source".*

**Author Reply:** We removed this specification, since it is related to the previous version of the system.

*What is the model of Photonic Professional system? Is it a GT2? Finally and most importantly, I believe that the DeScribe files (data, job, recipe etc) need to be included in the Extended data, in addition to the .stl files which are already included.*

**Author Reply: **We updated the info about our Nanoscribe system, that is the galvo update of GT, equivalent to a GT2. We also added an example of DeScribe outputs (containing full parameters for slicing and writing jobs) to the Extended Data. While doing this, we noticed a typo in the manuscript concerning the hatching distance (which was actually 0.2 instead of 0.3 as we previously wrote) and discrepancies in the reported laser power.

*Evaluation of actuation properties - “Peltri plate” – do the authors mean Peltier element? "repeatedly heated up to 70 °C and cooled down to room temperature" -  please include the number of heating / cooling cycles? "Measurements were taken using the image processing software of the instrument." - please provide the name and version of the software.*

**Author Reply: **We changed ‘plate’ to ‘element’ and added the rest of the information in the text.

*Simulations - in my opinion, the first paragraph of this section, with the exception of its first sentence, belongs in the Results and discussion section, rather than in the Methods section. How were the various parameters chosen? The authors mention is several places that xx is (...), "which we set to" yy (see Qn , Tni ). Similarly, "The LCE is modelled as a Linear Elastic Material with Young's Modulus E = 1 MPa, Poisson’s ratio ν ~ 0.5 (nearly incompressible), and density ρ = 1.2 g/cm3." - how were the values chosen? Could the authors explain their choice or provide suitable references?*

**Author Reply: **The first paragraph was added upon request of the editor to explain the reason why we used proprietary software for the simulations rather than an open source one. We do not think such statements are critical in the exposition of the methodology and therefore we prefer to place them in the methods section rather than in the discussion where they would unnecessary lengthen the part. The mechanical parameters and density values are intended as representative of this class of materials (chosen according to [S. Palagi, A. G. Mark, S. Y. Reigh, K. Melde, T. Qiu, H. Zeng, C. Parmeggiani, D. Martella, A. Sanchez-Castillo, N. Kapernaum, F. Giesselmann, D. S. Wiersma, E. Lauga, P. Fischer, Nat. Mater. 2016, 15, 647] – the reference has been added in the new version), and not necessarily accurate for the specific 3D-printed microstructures (for which accurate measurement of all properties was out of the scope of this work). We have chosen the transition-related parameters (Qn, Tni, gamma) to roughly match the behavior observed in our microstructures. Again, the simulations were not performed as a predictive tool, yet as a way to interpret the observed results and have an intuitive view of the shape-change behavior.

*Results and discussion The caption for Figure 1b is extremely confusing. There is no clear separation between the description of b) and the combined description of c) and d). I suggest rewriting as "a) Molecular structures of the liquid crystal elastomers (LCE) monomers employed. b) Indium-tin oxide (ITO) interdigitated electrodes: view of substrate. (c,d) Optical microscopy image of liquid crystals on substrate under cross polarizers, no bias applied (c) and with bias applied (d). e) Simulation of the distribution of the electric field generated by flat electrode, view along the plane (cross section)."*

**Author Reply:** To make it more clear, we rephrased the caption as “a) Molecular structures of the liquid crystal elastomers (LCE) monomers employed. b) Photograph of indium-tin oxide (ITO) interdigitated electrodes. c-d) Optical microscopy images of liquid crystals on substrate under cross polarizers no bias applied (c) and with bias applied (d). e) Simulation of the distribution of the electric field generated by flat electrode, view along the plane (cross section).”

*When discussing the LC formulation, the authors selected "the formulation comprising a 7:3 LC-MA:LC-DA mixture" as optimal. First of all, could the reasoning behind this choice be described in more detail. Secondly, the authors then refer to the "6:4 LC-MA:LC-DA" mixture as having "an excess of the difunctional LC", although the LC-DA is only 40% of the mixture. Could the authors reconsider the way they refer to the mixtures that have different ratios that the one deemed optimal?*

**Author Reply: **Following the advices of both reviewer 1 and 2, we rewrote the paragraph as “We tried several different compositions and found that the formulation comprising a molar ratio of 7:3 LC-MA:LC-DA gave the best results for our scope. In particular, when compared to different mixtures, the printed structures showed an appreciable resolution (Figure 2) and resulted soft enough to observe the actuation (as discussed later and depicted in Figure 4). Increasing the amount of monoacrylate compound (8:2 LC-MA:LC-DA) resulted in structures that looked softer and less defined (see Figure 2), while increase of the quantity of difunctional LC (6:4 LC-MA:LC-DA) gave rise to more rigid structures that did not actuate appreciably.”

*In Figure 2 - please include a) and b) in the caption, since the images list this.*

**Author Reply: **Added.

*In the caption of Figures 2-5 - please include a), b), c) etc before the corresponding text description, in a similar manner to the caption of Figure 1.*

**Author Reply: **Done.

*In Figure 4a and Figure 5a, is it possible to provide images with higher magnification, or at least to crop the outer black area? It is rather difficult to observe anything in the current panels.*

**Author Reply: **We followed the suggestion of the reviewer to make the picture more pleasant and we cropped part of the black area.

*As the authors mention, the alignment observed in the structures is sub-optimal. When addressing potential reasons, the authors do not mention the fact that an electric field induces a rather transient alignment, which might be lost during the DLW process. What is the fabrication time for the various structures? Have the authors investigated for how long after applying the electrical current do the mesogens stay aligned? The Methods section mentions that "The samples were then rapidly transferred to a Photonic Professional system" (after performing the alignment using the horizontal electric field). I believe that thoroughly characterizing the ability of the system to maintain its alignment over time, after the voltage source is removed, would be extremely beneficial, as the system could well lose its alignment over (relatively short) periods of time.*

**Author Reply:** We understand the concerns of the reviewer and indeed we did not perform such investigation on our system. While we do not know for certain the evolution of the orientation with time, because of the solid nature of the resist, we are confident that the alignment is retained for at least hours so to allow the printing process. Unfortunately, at the moment we won’t be able to perform such experiments anytime soon. We plan, however, to include this analysis in our follow up work on this subject.

*Regarding the simulations and experiments: the heating / cooling cycles were 70C / 25C in the experiments, but 80C / 40C. Why is that? Would it not be more beneficial to employ the same temperature? In the Methods section, the authors mention that "Tni is the nematic-to-isotropic transition temperature, which we set to 60°C". Why was this value chosen, and was it confirmed experimentally as well?*

**Author Reply: **While the simulations for the distribution of the electric field are helpful to explain the performances of flat electrodes, those concerning the response of LCEs are intended as support to clarify the expected actuation to readers not familiar with such materials. In this sense, they do not represent a full description of the system involved. In general, when such calculations are performed, they do not entail any molecular or atomistic details and thus the characteristic parameters (such as transition temperature, young modulus, and ordering) are external inputs. The 80/40 interval was set to represent the full deformation range across the transition temperature. This was set to 60 C since experimentally we noticed that the actuation started at around 50 C (both in the thin film and DLW printed structures) and was complete at 70 C as we mention in the text. For this reason, the interval of interest for the temperature response in simulations was chosen to be between 40C and 80C, which includes the one observed experimentally.

*Conclusions This sections should be expanded. Since this is a Methods article, I believe the Conclusions section could briefly reiterate the process flow and emphasise crucial steps.*

**Author Reply: **Such part was added as we agree that makes the article more effective for the readers.
